# Child mental health after institutional care in Azerbaijan: prevalence and correlates

**DOI:** 10.1186/s12889-026-26678-w

**Published:** 2026-03-11

**Authors:** Leyla Ismayilova, Narmin Guliyeva, Linyun Fu, Kamran Salayev, Emma Heidorn, Fuad Ismayilov, Parvin Muslumzada, Kerim Munir

**Affiliations:** 1 Crown Family School of Social Work, Policy, and Practice, The University of Chicago, Chicago, IL USA; 2 Department of Psychiatry, Azerbaijan Medical University, Baku, Azerbaijan; 3 Division of Newborn Medicine, Fetal-Neonatal Neuroimaging & Developmental Science Center, Boston Children’s Hospital, Boston, MA USA; 4 Department of Psychiatry, Azerbaijan Medical University; and National Mental Health Centre, Baku, Azerbaijan; 5 Department of Pediatrics and Department of Psychiatry and Behavioral Sciences, Boston Children’s Hospital, Harvard Medical School, Boston, MA USA

**Keywords:** Deinstitutionalization, Family reunification, Child mental health, Post-Soviet countries, Eurasia

## Abstract

**Purpose:**

To examine mental health symptoms and their associations with institutional and sociodemographic characteristics among school-aged children reunified with their families following deinstitutionalization in a country in the South Caucasus.

**Methods:**

This study reports cross-sectional findings from baseline data collected for a randomized clinical trial (ClinicalTrial.gov #NCT05396625). The sample included 434 children (ages 7–12) with histories of institutional placement and 305 caregivers (parents or kin-relatives) recruited in Azerbaijan following family reunification. Individual- and family-level socio-demographic characteristics were examined to assess their associations with mental health outcomes, as reported by children (self-esteem, symptoms of depression, post-traumatic stress disorder/PTSD) and by caregivers (Attention-Deficit/Hyperactivity Disorder/ADHD symptoms, and internalizing and externalizing behavioral problems). Mixed effects regression models were employed to account for clustering of children within families.

**Results:**

Over half (52%) of children with a history of institutional placement scored above the clinical cutoff for depressive symptoms on the Center for Epidemiologic Studies Depression Scale (CES-D), 37% for PTSD symptoms on the Child Revised Impact of Events Scale-8 (CRIES-8), and 36% for ADHD symptoms on the ADHD Rating Scale-IV. Caregiver reports on the Strengths and Difficulties Questionnaire (SDQ) indicated that 45% of children were at high or very high risk for emotional and behavioral problems, with internalizing (54.05%) more than twice as common as externalizing (24.54%). Middle school-aged children exhibited higher symptomatology across multiple mental health outcomes compared to those in elementary school, and children with special needs displayed elevated symptoms across all parent-reported outcomes. According to parent reports, girls showed fewer ADHD (-3.60, 95% CI: -5.80, -1.39) and externalizing (impulsivity, aggression, disruptive behaviors, -1.74, 95% CI: -2.60, -0.89]) but more internalizing symptoms (withdrawn, anxious, persistent sadness, low self-worth) than boys, while child-reported depression did not differ by gender. Lower socio-economic status (e.g., receipt of public assistance, perceived low SES, larger household size, residence outside the capital, and lower caregiver education) and maternal (vs. paternal or non-parental) reports were associated with higher parent-reported internalizing problems among children. Children who had stayed in boarding school-type institutions with weekend home returns showed fewer problems (-2.70, 95% CI: -5.10, -0.31), especially internalizing, than those from orphanages.

**Conclusion:**

A substantial proportion of children reunified with families following deinstitutionalization exhibit clinically significant mental health symptoms. Mental health symptomatology varied by age, gender, and socioeconomic disadvantage, with the latter exerting a stronger influence on parents’ perceptions than on children’s self-reports. These findings underscore the importance of integrating targeted preventive mental health programs with supportive family-based interventions that also address socio-economic factors, in order to better support this vulnerable population during and after the reintegration process.

## Introduction

Living in institutional care – including orphanages and other residential institutions – places children at a heightened risk for developing emotional and behavioral problems that can persist into adulthood, leading to increased risk for mental illness and suicide [20, 43]. Research conducted globally consistently demonstrates a higher prevalence of internalized and externalized mental disorders among children residing in such facilities [15, 29, 30]. Exposure to severe early-life neglect, as is often experienced in institutional rearing, has been shown to alter the development of neural circuitry involved in emotion regulation, resulting in an elevated risk of developing depression and anxiety disorders later in life [5]. Van IJzendoorn et al. found a dose–response association, with longer stays in the institution predicting larger developmental delays and deviations, the effect size being moderately large across all domains [41].

The countries of the former Soviet Union (fSU) and Eastern Europe have the highest rate of children in institutional care in the world [32]. Approximately 1.3 million children are raised in institutions in this region, and their mental health and longer-term developmental outcomes are of global public health concern [7, 13, 24]. While the importance of deinstitutionalizing childcare is universally recognized [45], its practical implementation faces multiple challenges, as each region and country operate within distinct socioeconomic, cultural, and service system contexts. Regional data indicates that a significant proportion of children in the region fail to thrive after reintegration, showing difficulties in educational, emotional and social adjustment [24]. In many cases, inadequate family support, poverty, and limited availability of and access to community-based services contribute to these challenges, placing children at heightened risk of re-entering institutional care [44].

Similar to other countries in the region, Azerbaijan has embraced the global deinstitutionalization movement, aiming to minimize the number of children in institutional care. As the complete closure of all institutions cannot be achieved instantaneously, it is more strategic to develop and implement evidence-based, contextually grounded deinstitutionalization and reintegration strategies that enable gradual and sustainable transitions [40]. Family reunification is widely regarded as the most optimal reintegration approach in Azerbaijan and across many fSU countries, given that many children in institutions have one or both living parents. Although deinstitutionalization reforms in the region began more than 20 years ago, research on this population remains limited–particularly with respect to children’s emotional and behavioral well-being following deinstitutionalization. A distinct feature of institutional childcare in fSU countries is that the majority of children are placed in institutions primarily for poverty-related reasons despite having living parents [8], underscoring the central role of socioeconomic and family-level factors in both the risk of institutional placement and the success of reintegration efforts [28]. While the benefits of family reintegration are widely acknowledged [45], little is known about the individual- and family-level factors in this region associated with more favorable emotional and behavioral adjustment of children following deinstitutionalization and reunification [2].

Advance prevention and the implementation of early interventions are among the six key global mental health priorities outlined in the U.S. National Institute of Health’s Grand Challenges in Global Mental Health [39]. Early prevention measures have the potential to mitigate the risk of developing mental disorders in adulthood and may concurrently influence a range of psychosocial outcomes [3]. Skinner et al. demonstrated that preventive interventions targeting adolescents with subthreshold symptoms may be more effective in reducing the prevalence of mental disorder than universal interventions delivered to the general population [36]. Thus, individuals with a history of institutional placement represent a specific subgroup with shared risk factors for mental health disorders who may particularly benefit from evidence-based interventions. At the same time, many existing mental health interventions developed in the U.S. depend heavily on highly skilled clinicians and extensive infrastructure, making them not readily adaptable to the context of developing countries with limited financial and human mental health resources. Although each country in the fSU region has its own mental health care systems and institutional childcare patterns, they share many socio-economic and cultural characteristics, including ongoing efforts to restructure their health, education, and social care systems in the aftermath of the Soviet legacy. Mental health, in particular, has gained increased attention in recent years; however, professional mental health services remain highly limited. Against this backdrop, it is critical to identify high-risk populations and examine the demographic and socioeconomic correlates of child mental health problems, in order to inform and strategically target mental health preventive and treatment efforts.

This study examines the prevalence of mental health symptoms and their associations with demographic and socioeconomic factors among children who have been reunited with their families after a history of institutional placement in Azerbaijan, a middle-income country in the South Caucasus. Using baseline data from a randomized clinical trial, the paper aims to:

- Assess the level of mental well-being and the prevalence of mental health symptoms among school-age children who have undergone institutional placement and subsequent reunification with their biological families in Azerbaijan;
- Examine discrepancies in child mental health reported by the children themselves and their caregivers;
- Identify demographic and socioeconomic factors that are associated with mental health symptoms among reunified children.

The insights gained from this study hold the potential to inform the development of contextually specific and culturally tailored mental health interventions for children with a history of institutional placement in Azerbaijan and other nations in the Eurasian region.

## Methods

This study uses baseline data from a randomized clinical trial with deinstitutionalized children in Azerbaijan funded by the Eunice Kennedy Shriver National Institute of Child Health and Human Development (NICHD) and Fogarty International (R01HD099847). In accordance with NIH ethical standards for clinical research, the study was approved by the Ethical Committee of Azerbaijan Medical University and the Institutional Review Board (IRB) at the University of Chicago (IRB20-1018). The trial is registered with the ClinicalTrials.gov database (NCT05396625), with the registration completed on May 19, 2022.

### Study site

The study took place in Azerbaijan, a middle-income post-Soviet country located in the Caucasus region. Most institutions in Azerbaijan are publicly run and provide not only accommodation, clothing and food but also education to the children residing within them, offering an opportunity to low-income families to secure essential care and schooling for their children when they are unable to provide these themselves. Institutions also differ in terms of the length and pattern of children’s stay: in some cases, children reside there throughout the entire academic year and return to their parents only during holidays and school breaks, while in others they live in the institution during the school week and spend weekends at home.

### Study population

A total of 434 children and 305 caregivers were recruited from the three largest cities in Azerbaijan: Baku, Sumgait, and Ganja. The eligibility criteria for child participants included: (1) aged between 7 and 12 years; (2) a history of institutional placement (i.e., having lived in an orphanage or ‘internat/boarding school’ for at least one month); and (3) reunification with their primary caregiver (either a biological parent or kin relative). For caregiver participants, the criteria included: (1) being the child’s biological parent or kin relative; (2) having been reunified with the child from an institution; and (3) being at least 18 years old. Children and caregivers with significant cognitive or mental health impairment (e.g., severe developmental disorder, psychosis) that might have interfered with either their ability to benefit from the prevention program or to participate safely were excluded from the study. Among screened participants, ten were found ineligible due to being outside the eligible age range, and twelve did not meet criteria due to a lack of institutional care history, including three who were siblings of enrolled participants and had not personally lived in an institution. Among eligible families, two declined participation, primarily due to lack of parental time. Additionally, two eligible children were excluded due to impairment—one with a speech disorder and one with cognitive impairment associated with a Down syndrome diagnosis. All eligible and consenting children in the family were allowed to participate.

### Procedure

Informed consent and assent procedures were conducted prior to the baseline assessments by data collectors, who were clinical psychologists by training. Children completed the assent process separately to avoid coercion.

Child and caregiver self-reported data were collected by data collectors in the Azerbaijani language using Qualtrics, a cloud-based survey software. Surveys included child-reported and parent-reported versions. Assessments were conducted in the field office and took approximately 40 to 60 min. Participants were informed they could decline or withdraw from the study at any time point without penalty. Parents provided reports of child behavior at home (Strengths and Difficulties Questionnaire/SDQ [19], 25 items; ADHD–IV Rating Scale [14], 18 items). All data collectors received structured training in the ethical conduct of research from the study investigators. More details are provided in a manuscript describing the study protocol (Ismayilova et al., under review).

### Measures

Socio-demographic variables at both child and family levels, as well as standardized measures for assessing children’s mental health outcomes—including self-esteem [17], depression [16], PTSD [31], ADHD [14], and SDQ [19]—were utilized, as detailed in Table 1.

**Table 1:** Description of measures

Measures	Description	Variables	Rater
Socio-demographic characteristics	*Child-level characteristics*: child’s gender, age, birth order, study site, presence of special needs, and age at entry to and exit from the institution. All child socio-demographic variables are reported by caregiver, with the exception of gender. *Family- and caregiver-level characteristics: caregiver’s type*, gender, age, marital status, education level, household size, monthly household income, employment status, perceived social economic status (SES), and an 8-item economic hardship index (assessing deprivation in food, medicine, heating, clothing, etc.), rated on a scale of *Never (0)*,* Sometimes (1)*,* Constantly (2)*.	Binary variables were created for most socio-demographic variables at the child and family levels, except for location (Baku, Sumgait, and Ganja), birth order (first, middle, youngest), caregiver type (mother, father, and other such as grandparent, uncle/aunt, etc.), and the Economic Hardship Index (averaged score).	Child/Parent
Child Mental Health (*reported by children*)			
The Tennessee Self-Concept Scale (TSCS-2) - Short Form	This standardized self-report instrument was developed by Fitts and Warren [16] to assess a child’s general self-perception. The short version comprises 20 items (e.g., *I usually feel good about myself. I often worry about not being good enough*). Each item is rated on a five-point Likert scale, ranging from 1 (Always False) to 5 (Always True) [16].	The scores from the 20 items were summed to yield a total self-concept, often used as an indicator of self-esteem, with higher scores indicating more positive and lower scores reflecting a more negative self-perception.	Child
The Center for Epidemiological Studies Depression Scale for Children (CES-DC)	This standardized 20-item scale was designed to screen for depression in children and adolescents ages 6–17 [15]. Each item reflects the frequency of depressive symptoms experienced in the past week (e.g., *I felt like crying*,* I felt that no one really loved me*). Each item is rated on a 4-point Likert scale ranging from 0 (Not at All) to 3 (A Lot) [15].	Two outcome variables were calculated: total depression scores and a binary variable determined by the cutoff scores. For the total scores, higher scores indicate a greater risk of depression, and lower scores suggest a lower risk. For creating binary variable, a cutoff score of 15 was used to identify children who are potentially depressed.	Child
The Child Revised Impact of Events Scale (CRIES-8)	This brief self-report screening tool is designed to assess posttraumatic stress reactions in children and adolescents [26]. The version consists of 8-items, divided into two symptoms clusters: *Intrusion* subscale (4 items reflecting re-experiencing of the traumatic event; e.g., *I have bad dreams about it*,* Pictures of what happened pop into my mind.*) and *Avoidance* subscale (4 items reflecting attempts to avoid trauma-related thoughts, feelings, or reminders; *I try not to think about what happened*,* I stay away from people*,* places*,* or things that remind me of it.*). This scale is a four-point Likert scale, including, not at all (0), rarely (1), sometime (3), and often (5) [25]. A cutoff score of 17 or higher is used to screen for potential PTSD in children.	Four outcome variables were calculated: total trauma scores, intrusion subscale scores, avoidance subscale scores, and a binary variable determined by the cutoff scores. A total score of 17 or higher served as the clinical cutoff for dichotomizing the variable. For both the total and subscale scores, higher values indicate greater PTSD symptom severity, whereas lower values reflect fewer symptoms.	Child
Child Mental Health (*reported by parents*)			
ADHD Rating Scale-IV	It is a tool for assessing the frequency and severity of symptoms of Attention Deficit Hyperactivity Disorder (ADHD) in children and adolescents [13]. The instrument consists of 18 items, divided into two subdomains: *inattention and hyperactivity-impulsivity*. Each item is rated on a scale ranging from 0 (never or rarely) to 3 (very often).	Both binary variable and total score variable were created. A cut-off score of 19 was adopted as an indicator of heightened risk for ADHD (Coghill & Seth, 2015). Three outcome variables were calculated: *total ADHD scores*,* inattention scores*,* and hyperactivity-impulsivity scores*; the latter two were the subscales.	Parent
The Strengths and Difficulties Questionnaire (SDQ)	It was developed by Goodman, includes 25 items completed by caregivers to screen for emotional and behavioral problems in children [17]. Each item is rated on a three-point Likert scale, consisting of 0 (Not True), 1 (Somewhat True), and 2 (Certainly True). This scale contains five subscales: *emotional problems*,* conduct problems*,* hyperactivity*,* peer problems and prosocial behaviors*. Alternatively, the scale can be divided into two subscales: internalizing scores and externalizing scores.	A found-band categorization (newer approach) and three-band categorization (older approach) of SDQ scores have both been both utilized for comparison purpose. Three additional outcome variables were calculated: *total SDQ difficulty scores*,* internalizing scores*,* and externalizing scores*. Higher scores indicate more severe problems, whereas lower scores denote lesser risk of these problems.	Parent

These standardized measures have not been formally validated in the Azerbaijani context, but they were pilot tested prior to the study

### Statistical analyses

The statistical analyses were conducted using STATA 17. To estimate the prevalence of mental health problems, frequency analysis was employed using the cutoff scores for each child mental health scale. The prevalence of co-occurring mental health challenges that exceeded clinical threshold has also been presented to illustrate the comorbidity among children who have been deinstitutionalized. We defined co-occurring multiple challenges as meeting the clinical threshold on two or more mental health measures. The prevalence of coexisting conditions was calculated within the subgroup of children with multiple challenges, defined as the proportion of these children who also exceeded the clinical threshold for each specific condition (depression, PTSD, ADHD, internalizing problems, and externalizing problems).

Given the nested structure of the data, with children clustered within families, mixed effects models were employed to account for family-level clustering [37]. These models enable the examination of variability at family levels. Individual-level and family-level socio-demographic variables in these models were included to explore their association with each mental health outcome, considering family clustering effects. Continuous outcome variables (e.g., scores from the self-esteem, ADHD, and SDQ scales) were analyzed using mixed effect models with a linear (Gaussian) link function. Mixed effects models with a logistic function were used for binary outcomes variables created using clinical cutoffs (e.g., depression and PTSD). Likelihood ratio tests were conducted to compare the multilevel model with the ordinary least squares (OLS) regression model.

## Results

### Participant socio-demographic characteristics

As presented in Table 2, the study encompassed 434 children and 305 of their caregivers. Among the child participants, boys made up a slight majority (61.52%) and nearly half were firstborns in their families. The age distribution showed that 54.38% were pre-adolescents (10–12 years old), while 45.62% were elementary schoolers (7–9 years old). A significant portion, 84.83%, previously resided in a general boarding school. The mean age of children entering the institution was 6.8 years (SD = 2.18), which corresponds to the typical school entry age. The average duration of institutional care was 2.88 years (SD = 2.39). A total of 67.25% of the children slept at home every night, while 32.75% spent at least some nights at school, including those who slept at school every night, throughout the entire week, or on weekdays. Geographically, the children were almost evenly distributed among the three study sites. A very small proportion of children had lost a mother (2.07%) or father (4.38%), and 5.30% of children had special needs (e.g., physical, sensory, or mental disability) reported by caregivers.

**Table 2:** Demographic characteristics of study participants

Social-demographic characteristics of the sample	*N*	%	Mean	SD
Child participants (*N* = 434)				
Child’s age in years (min 7 – max 12)	434		9.58	1.67
Elementary school graders (ages 7–9)	198	45.62%		
Pre-adolescent (ages 10–12)	236	54.38%		
Child’s gender				
Girl	167	38.48%		
Boy	267	61.52%		
Birth order				
First born	199	45.75%		
Middle born	73	16.78%		
Last child	163	37.47%		
Age of entry to the institution	434		6.8	2.18
Duration of institutionalization	434		2.88	2.38
Type of previous institution				
Orphanage	50	11.49%		
Internat (a boarding school–like institution)	369	84.83%		
SOS-Children’s Village and other	16	3.68%		
Frequencies of sleeping at home				
Staying at home every night	271	67.25%		
Sleep at school during the week and at home during the weekend	93	23.08%		
Sleep at school during the entire week	17	4.22%		
Staying at school most of the year	22	5.46%		
Children whose biological mother is not alive	9	2.07%		
Children whose biological father is not alive	19	4.38%		
Children who have special needs	23	5.30%		
Caregiver participants (*N* = 305)				
Caregiver’s age in years	305		38.53	9.77
Caregiver’s gender				
Female	282	92.46%		
Male	23	7.54%		
Participating caregiver				
Mother	243	79.67%		
Father	15	4.92%		
Grandmother	30	9.84%		
Other (grandfather, aunt, uncle, stepparent)	17	5.57%		
Residence				
Baku (capital)	152	35.02%		
Sumgait	137	31.57%		
Ganja	145	33.41%		
Household size	304		4.54	1.71
Number of children per caregiver in the study				
One child	198	64.92%		
Two children	87	28.52%		
Three children	18	5.90%		
Four children	2	0.66%		
Marital status				
Married (in a civil or religious married marriage)	174	57.05%		
Other (single, divorced, windowed, second wife, other)	131	42.95%		
Monthly income				
0–300₼ AZ Manat (176 USD) per month	135	44.52%		
≥ 301₼ AZ Manat (176 USD) per month	167	55.48%		
Religion (Muslim)	304	99.67%		
Education Level				
Primary school (grades 1–4)	20	6.56%		
General (basic) secondary education (grades 5–9)	83	27.21%		
Full (complete) secondary education (grades (10–11)	97	31.8%		
Vocational school or specialized secondary education (college, technikum)	74	24.26%		
Higher education	31	10.16%		
Caregiver self-perception of economic situation				
Very bad	39	12.79%		
Bad	91	29.84%		
Average	166	54.43%		
Good	7	2.3%		
Very good	2	0.66%		
Economic Hardship Index, *mean* (score 0–2)	305		1.12	0.56
Employment status				
Employed (full time or part-time)	144	47.21%		
Other status (unemployed, retired, disability status)	161	52.79%		
Receiving public assistance				
Targeted social assistance	26	8.52%		
Disability benefits for my child	9	2.95%		
Disability benefits for myself or other family members (e.g., husband)	14	4.59%		
Alimonies	21	6.89%		
Retirement pension	11	3.61%		
Benefits for war veterans and their families	6	1.97%		
Benefits for refugees and internally displaced (IDP) status	8	2.62%		
Child benefits	3	0.98%		

The majority of caregivers were female (92.46%) and biological mothers (79.67%). The mean age was 38.53 years (SD = 9.77), and the mean household size was 4.54 (SD = 1.7). Over a third of families (35.08%) had more than one child participating in this study. Over half (57.05%) were in either a civil or religious marriage. Regarding educational attainment, 34.1% of caregivers had not completed full secondary education (11 years of schooling). Concerning their economic situation, 42.95% perceived their social-economic status (SES) as bad or very bad, and 44.52% reported a monthly income below 300 Azerbaijani Manat/AZN (approximately $175 USD). This is slightly below the current national minimum wage of 400 AZN (about $235 USD) per month. In addition, the mean economic hardship index score was 1.12 (SD = 0.56), indicating that over the past 12 months families often struggled to meet at least one of nine essential needs, with medical care being the most commonly lacking resource (84.93%). Over a half of caregivers (52.79%) were outside of the labor force (full-time or part-time employment), including those who were unemployed, retired, or unable to work due to disability. Furthermore, 39.34% reported receiving public assistance.

### Prevalence and co-occurrence of child mental health problems

#### Reported by children

As shown in Table 3, the mean self-esteem score was 82.54 (SD = 11.33). A significant proportion of children with a history of institutionalization exhibited various mental health challenges. Specifically, 52% of the children were identified as having depression scores above the clinical range, and 36.53% showed signs of PTSD above the clinical range. The mean score on the avoidance subscale (6.64, SD = 7.21) was higher than the mean score on the intrusion subscale (4.81, SD = 5.73).

**Table 3:** Prevalence of child mental health variables (*N* = 434)

Child Mental Health	Cronbach’s α	*N*	%	Mean	SD	Theoretical Scale Range
Reported by Children						
TSCS-2 Self-Concept Score, *mean*	0.80	419		82.54	11.33	20–100
CES-DC Depression Score, *mean*	0.85	425		16.48	10.89	0–60
Above clinical range (≥ 15), %		221	52%			
CRIES-8 Trauma Score, *mean*	0.91	427		11.42	11.80	0–40
Intrusion subscale, *mean*	0.87	429		4.81	5.73	0–20
Avoidance subscale, *mean*	0.89	429		6.64	7.21	0–20
Above clinical range (≥ 17), %		156	36.53%			
Reported by Parents						
Strengths and Difficulties Questionnaire (SDQ)						
Total Difficulty Score, *mean*	0.80	430		15.67	7.00	0–40
Four-band categorization						
Close to average		174	40.70%			
Slightly raised		64	14.88%			
High		63	14.65%			
Very high		129	30.00%			
Three-band categorization						
Normal		174	40.47%			
Borderline		64	14.88%			
Abnormal		192	44.65%			
Emotional problems, *mean*	0.71	434		4.82	2.73	0–10
Four-band categorization						
Close to average		154	35.48%			
Slightly raised		55	12.67%			
High		96	22.12%			
Very high		129	29.72%			
Three-band categorization						
Normal		154	35.48%			
Borderline		55	12.67%			
Abnormal		225	51.84%			
Conduct problems, *mean*	0.67	434		2.83	2.27	0–10
Four-band categorization						
Close to average		210	48.39%			
Slightly raised		70	16.13%			
High		101	23.27%			
Very high		53	12.21%			
Three-band categorization						
Normal		210	48.39%			
Borderline		70	16.13%			
Abnormal		154	35.48%			
Hyperactivity sub-scale, *mean*	0.66	433		4.68	2.69	0–10
Four-band categorization						
Close to average		270	62.36%			
Slightly raised		87	20.09%			
High		29	6.70%			
Very high		47	10.85%			
Three-band categorization						
Normal		270	62.36%			
Borderline		52	12.01%			
Abnormal		111	25.64%			
Peer problems, *mean*	0.38	434		3.40	1.84	0–10
Four-band categorization						
Close to average		158	36.41%			
Slightly raised		81	18.66%			
High		86	19.82%			
Very high		109	25.12%			
Three-band categorization						
Normal		158	36.41%			
Borderline		81	55.07%			
Abnormal		195	44.93%			
Prosocial behaviors, *mean*	0.73	435		8.06	2.26	0–10
Four-band categorization						
Close to average		294	67.59%			
Slightly lowered		47	10.80%			
Low		32	7.36%			
Very low		62	14.25%			
Three-band categorization						
Normal		373	85.75%			
Borderline		24	5.52%			
Abnormal		38	8.74%			
Internalizing score, *mean*	0.71	435		9.31	5.20	0–20
Four-band categorization						
Close to average		53	12.24%			
Slightly raised		146	33.72%			
High		112	25.87%			
Very high		122	28.18%			
Externalizing score, *mean*	0.78	432		7.47	4.46	0–20
Four-band categorization						
Close to average		215	49.77%			
Slightly raised		111	25.69%			
High		65	15.05%			
Very high		41	9.49%			
ADHD RS-IV score (continuous)						
ADHD Total Score, *mean*	0.92	427		16.16	11.41	0–54
Inattention sub-scale, *mean*	0.90	431		7.86	6.30	0–27
Hyperactivity-Impulsivity subscale, *mean*	0.86	430		8.31	6.09	0–27
ADHD RS-IV above clinical range (binary), %						
Total score above clinical range (≥ 19)		153	35.83%			
Total score above 90% percentile (≥ 32)		45	10.54%			
Inattention subscale above clinical range (≥ 10)		156	35.37%			
Hyperactivity-Impulsivity subscale above clinical range (≥ 10)		171	38.78%			

*TSCS*-*2* Tennessee Self-Concept Scale, Second Edition, *CES*-*DC* Center for Epidemiological Studies Depression Scale for Children, *CRIES*-*8* Children’s Revised Impact of Event Scale – 8 item version, *SDQ* Strengths and Difficulties Questionnaire, *ADHD* *RS*-*IV* ADHD Rating Scale IV

#### Reported by parents

According to the parent reports on SDQ Total Difficulty scale, 44.65% of children fell within the ‘high’ or ‘very high’ risk categories for emotional and behavioral problems. Specifically, 54.05% were rated at ‘high’ or ‘very high’ risk for internalizing problems (emotional and peer problems) and 24.54% for externalizing problems (hyperactivity and conduct problems). Over two thirds (67.59%) demonstrated prosocial behaviors close to the average range. In addition, over one-third of children (35.83%) were reported by parents to exhibit clinically significant ADHD symptoms, with relatively comparable proportions scoring above the clinical threshold on the hyperactivity–impulsivity (38.78%) and inattention (35.37%) subscales.

#### Comorbidity

Among all participating children, 81.39% exhibited at least one mental health challenge above the clinical threshold. Of these children, 59.8% faced multiple challenges, with an average of 1.98 challenges per child (SD = 1.43, range = 0–5). Within this group, the prevalence of coexisting conditions indicated the proportion of children who exceeded the clinical threshold for each condition in combination with at least one other problem: 86.21% for depression, 86.58% for PTSD, 97.14% for ADHD, 84.83% for internalizing problems, and 96.88% for externalizing problems. For example, 86.21% of children above the clinical threshold for depression also met the threshold for at least one other condition, meaning only about 14% had depression alone.

### Individual and family sociodemographic correlates of child mental health

#### Child-reported mental health (self-esteem, depression, and trauma)

Table 4 presents the estimates from mixed effects regression models examining child- and family-level factors associated with child-reported self-esteem scores, as well as symptoms of depression and trauma above the clinical threshold.

**Table 4:** Multilevel regression and logistic regression models for child reported mental outcomes and child/family characteristic

	Self-concept (Coeff, 95% CI) n=411	Depression Symptoms(OR, 95% CI) n=417	PTSD Symptoms(OR, 95% CI) n=419
Intercept	81.15*** [73.12, 89.20]	0.50 [0.07, 3.63]	0.21 [0.03, 1.76]
Child Characteristics			
Preadolescent (aged 10-12)	-2.11* [-4.15, -0.07]	1.56^+^ [0.92, 2.64]	2.20** [1.21, 3.98]
Girl	2.47* [0.48, 4.47]	0.64^+^ [0.37, 1.09]	0.98 [0.57, 1.69]
Birth order (*ref. *first child)			
Middle child	-3.31* [-6.08, -0.54]	1.26 [0.61, 2.58]	1.06 [0.51, 2.23]
Last child (youngest)	-1.52 [-3.60, 0.56]	1.03 [0.60, 1.78]	1.09 [0.61, 1.96]
Having special needs	-1.39 [-5.86, 3.08]	0.54 [0.17, 1.75]	2.45 [0.72, 8.33]
Years spent in institution	-0.12 [-0.69, 0.45]	1.07 [0.92, 1.24]	1.00 [0.85, 1.16]
Type of institution (*ref. *orphanage)			
Boarding school (Internat)	4.01^+^ [-0.01, 8.04]	1.37 [0.31, 3.65]	0.84 [0.31, 2.32]
SOS village and other	-6.66^+^[-13.64, 0.32]	2.28 [0.39, 13.22]	1.74 [0.31, 9.62]
Family or Caregiver Characteristics			
Caregiver age	0.07 [-0.09, 0.23]	1.01 [0.97, 1.05]	0.99 [0.95, 1.03]
Caregiver type (*ref. *mother)			
Father	-1.69 [-6.79,3.42]	1.57 [0.45, 5.51]	1.07 [0.30, 3.82]
Other	-4.15^+^ [-8.30, 0.01]	1.44 [0.51, 4.00]	1.20 [0.41, 3.50]
Caregiver married	0.49 [-2.08, 3.05]	0.56^+^ [0.30, 1.05]	0.82 [0.43, 1.54]
Caregiver completed secondary education	0.42 [-2.05, 2.90]	1.05 [0.58, 1.92]	1.42 [0.76, 2.67]
Caregiver employed	1.45 [-0.94, 3.83]	0.73 [0.41, 1.30]	1.73 [0.94, 3.20]
Caregiver perceived low SES	-1.08 [-3.53, 1.38]	1.07 [0.59, 1.95]	1.27 [0.68, 2.39]
Economic hardship index	-0.67 [-2.88, 1.54]	1.23 [0.72, 2.12]	0.71 [0.41, 1.25]
Family receiving public assistance	-3.35** [-5.83, -0.86]	1.76^+^ [0.96, 3.21]	1.71^+^ [0.92, 1.18]
Household size	0.38 [-0.32, 1.08]	0.99 [0.83, 1.17]	1.10 [0.92, 1.32]
City (*ref. *Baku)			
Sumgait	-3.21* [-6.20, -0.22]	0.99 [0.48, 2.05]	2.31* [1.08, 4.94]
Ganja	-5.93*** [-9.15, -2.71]	0.65 [0.30, 1.43]	0.44^+^ [0.19, 1.03]
Variance components			
Family-level (s.e.)	10.05	0.82	0.89
ICC	0.35	0.27	0.29

*OR* odds ratios

^+^*p*<0.1, *p<0.05, **p<0.01, ***p<0.001

##### Child-level factors

Controlling for other socio-demographic variables and the effect of family clustering, child’s older age was consistently associated with elevated mental health symptomatology. Compared to elementary school-age children (aged 7–9), preadolescents (aged 10–12) reported significantly lower self-esteem (-2.11, 95% CI: -4.15, -0.07), along with increased odds of trauma symptoms above the clinical range (OR = 2.20, 95% CI: 1.21, 3.98). Compared to first-born children, middle-born children reported lower self-esteem scores (-3.31, 95% CI: -6.08, -0.54).

##### Family-level factors

Children living outside of the capital city Baku also consistently showed elevated mental health symptomatology, specifically lower self-esteem (-5.93, 95% CI: -9.15, -2.71 for children in Ganja) and higher odds of the clinically significant trauma symptoms (OR = 2.31, 95% CI: 1.08, 4.94 for children in Sumgait). No regional differences were detected in child-reported depressive symptoms. At the family level, children from families receiving public assistance reported significantly lower self-esteem (-3.35, 95% CI: -5.83, -0.86).

##### Variance explained at the family level

The models indicated that 35% of the variance in child-reported self-esteem scores, 27% in depression above the clinical range, and 29% in trauma symptoms could be explained at the family level.

### Parent-reported child mental health (ADHD and SDQ)

Table 5 displays the coefficients from mixed effects regression models adjusting for socio-demographic variables and family clustering effects for parent-reported ADHD scale (total score and the two subscales—inattention and hyperactivity-impulsivity) and SDQ scale (total difficulties score and the two subscales—internalizing and externalizing problems).

**Table 5:** Multilevel/OLS regression models for parent-reported child mental outcomes and child/family characteristics

	ADHD Total Score(Coeff, 95% CI) n=418	ADHD Inattention Subscale(Coeff, 95% CI) n=422	ADHD Hyperactivity-Impulsivity Subscale (Coeff, 95% CI)n=421	SDQ Total Difficulties Score(Coeff, 95% CI) n=421	SDQ Internalizing Subscale (Coeff, 95% CI) n=424	SDQ Externalizing Subscale(Coeff, 95% CI) n=423
Intercept	11.06** [3.15, 18.97]	3.69 [-0.68, 8.07]	7.60*** [3.39, 11.80]	12.08*** [7.29, 16.87]	6.46*** [2.95, 9.96]	6.18*** [3.12, 9.25]
Child Characteristics						
Preadolescent (aged 10-12)	2.27* [0.03, 4.50]	1.43* [0.19, 2.67]	0.83 [-0.36, 2.02]	1.17^+^ [-0.10, 2.45]	1.55** [0.61, 2.49]	0.24 [-0.63, 1.11]
Girl	-3.60** [-5.80 -1.39]	-1.52* [-2.74, -0.29]	-2.11*** [-3.28, -0.93]	-1.15^+^ [-2.40, -0.10]	0.98* [0.05, 1.90]	-1.74*** [-2.60, -0.89]
Birth order (*ref. *first child)						
Middle child	-0.85 [-3.99, 2.30]	-1.33 [-3.07, 0.40]	0.48 [-1.17, 2.14]	-1.15 [-2.88, 0.58]	-1.12^+^ [-2.41, 0.16]	-0.34 [-1.54, 0.86]
Last child (youngest)	0.46 [-1.92, 2.83]	-0.39 [-1.70, 0.93]	0.76 [-0.50, 2.03]	-0.28 [-1.60, 1.04]	-0.64 [-1.62, 0.33]	0.04 [-0.88, 0.97]
Having special needs	7.46** [2.57, 12.36]	3.51* [0.79, 6.23]	3.78** [1.22, 6.34]	5.12*** [2.35, 7.89]	2.74** [0.69, 4.79]	3.05** [1.18, 4.92]
Years spent in institution	0.01 [-0.59, 0.61]	-0.07 [-0.40, 0.26]	0.08 [-0.24, 0.40]	0.05 [-0.31, 0.40]	0.02 [-0.24, 0.28]	0.04 [-0.20, 0.27]
Type of institution (*ref.* orphanage)						
Boarding school (Internat)	-2.21 [-6.09, 1.66]	-0.76 [-2.89, 1.37]	-1.47 [-3.51, 0.57]	-2.70* [-5.10, -0.31]	-2.16* [-3.89, -0.43]	-0.72 [-2.21, 0.77]
SOS village and other	5.31 [-1.50, 12.12]	1.28 [-2.39, 4.96]	3.29^+^ [-0.33, 6.91]	0.09 [-3.88, 4.05]	-1.99 [-4.89, 0.92]	1.56 [-1.02, 4.14]
Family or Caregiver Characteristics						
Caregiver age in years	0.12 [-0.03, 0.28]	0.07 [-0.02, 0.16]	0.05 [-0.04, 0.13]	0.11* [0.01, 0.20]	0.03 [-0.04, 0.10]	0.06* [0.00, 0.13]
Caregiver type (*ref. *mother)						
Father	-3.75 [-8.50, 1.00]	-1.51 [-4.14, 1.13]	-2.22^+^ [-4.76, 0.32]	-5.04** [-8.01, -2.08]	-4.02*** [-6.19, -1.85]	-2.26* [-4.12, -0.41]
Other	-2.73 [-6.82, 1.35]	-0.81 [-3.07, 1.46]	-1.85^+^ [-4.03, 0.33]	-1.96 [-4.42, 0.49]	-1.83* [-3.63, -0.03]	-0.48 [-2.06, 1.10]
Caregiver married	0.08 [-2.38, 2.55]	0.45 [-0.92, 1.81]	-0.47 [-1.78, 0.85]	-0.23 [-1.73, 1.28]	-0.03 [-1.13, 1.07]	-0.08 [-1.04, 0.88]
Caregiver completed secondary education	-1.60 [-3.97, 0.77]	-0.91 [-2.22, 0.41]	-0.63 [-1.89, 0.63]	-1.51* [-2.97, -0.05]	-0.72 [-1.79, 0.34]	-0.83^+^ [-1.75, 0.09]
Caregiver employed	-1.40 [-3.69, 0.89]	-0.21 [-1.48, 1.06]	-1.17^+^ [-2.39, 0.05]	-0.25 [-1.65, 1.14]	0.21 [-0.81, 1.23]	-0.23 [-1.12, 0.66]
Caregiver perceived low SES	2.95* [0.58, 5.32]	1.75** [0.44, 3.07]	1.14^+^ [-0.12, 2.41]	1.92* [0.47, 3.37]	0.95^+^ [-0.11, 2.00]	1.13* [0.21, 2.06]
Economic hardship index	-0.21 [-2.37, 1.94]	0.23 [-0.95, 1.42]	-0.46 [-1.60, 0.69]	-0.12 [-1.42, 1.18]	0.59 [-0.36, 1.54]	-0.67 [-1.50, 0.16]
Family receiving public assistance	0.62 [-1.76, 2.99]	0.70 [-0.61, 2.02]	-0.01 [-1.27, 1.25]	-0.09 [-1.55, 1.36]	0.43 [-0.62, 1.49]	-0.25 [-1.17, 0.67]
Household size	0.34 [-0.35, 1.02]	0.27 [-0.12, 0.65]	0.08 [-0.29, 0.44]	0.32 [-0.09, 0.74]	0.40* [0.09, 0.70]	0.09 [-0.18, 0.36]
City (*ref. *capital city Baku)						
Sumgait	3.44* [0.54, 6.34]	1.36^+^ [-0.24, 2.95]	2.03** [0.50, 3.57]	2.46** [0.68, 4.25]	1.72** [0.43, 3.01]	0.98^+^ [-0.14, 2.11]
Ganja	0.43 [-2.69, 3.56]	-0.65 [-2.39, 1.08]	1.13 [-0.54, 2.80]	1.09 [-0.81, 2.98]	-0.78 [-2.17, 0.61]	0.88 [-0.34, 2.10]
Variance components						
Family-level (s.e.)	n/a	n/a	n/a	3.59	1.94	n/a
ICC	n/a	n/a	n/a	0.21	0.18	n/a

Likelihood ratio tests were conducted to compare the multilevel model with the ordinary least squares (OLS) regression model

^+^*p*<0.1, *p<0.05, **p<0.01, ***p<0.001

#### Child-level factors

Among child characteristics, age, gender, and special needs status consistently showed associations across all mental health outcomes reported by parents. In comparison to elementary school children, preadolescents exhibited significantly higher symptoms of ADHD (2.27, 95% CI: 0.03, 4.5), including symptoms of inattention (1.43, 95% CI: 0.19, 2.67) as well as elevated SDQ internalizing problems (1.55, 95% CI: 0.61, 2.49). In comparison to boys, parents reported that girls had significantly fewer ADHD symptoms (-3.60, 95% CI: -5.80, -1.39) —encompassing both symptoms of inattention and hyperactivity-impulsivity — along with elevated internalizing problems (0.98, 95% CI: 0.05, 1.90) and lower externalizing problems (-1.74, 95% CI: -2.60, -0.89). Parents of children with special needs reported significantly higher scores for all mental health variables, including ADHD (7.46, 95% CI: 2.57, 12.36) and all SDQ scores (5.12, 95% CI: 2.35, 7.89).

Compared to children who had previously lived in orphanages, where they typically reside year-round, children with a prior placement in local boarding schools (‘internats’, where they usually stay overnight during the school week only) were reported by parents as having significantly lower SDQ total difficulties scores (-2.70, 95% CI: -5.10, -0.31), particularly lower levels of internalizing problems (-2.16, 95% CI: -3.89, -0.43).

#### Caregiver characteristics

At the family level, older caregivers reported higher SDQ total difficulties among children (0.11, 95% CI: 0.01, 0.20) and SDQ externalizing problems (0.06, 95% CI: 0.00, 0.13). Compared to mothers, fathers participating in the study as main caregivers reported fewer SDQ total difficulties (-5.04, 95% CI: -8.01, -2.08), including fewer internalizing (-4.02, 95% CI: -6.19, -1.85) and externalizing problems (-2.26, 95% CI: -4.12, -0.41). Non-parental caregivers (e.g., grandparents, aunts) were also less likely to report internalizing problems among children (-1.83, 95% CI: -3.63, -0.03) compared to reports from mothers.

#### Socio-economic status

Socio-economic status of the family was linked to mental health reports of children in various ways. Caregivers’ perceived low socioeconomic status (SES) was linked higher ADHD symptoms (2.95, 95% CI: 0.58, 5.32) —particularly symptoms of inattention (1.75, 95% CI: 0.44, 3.07) —and increased reports of SDQ total difficulties (1.92, 95% CI: 0.47, 3.37), specifically externalizing problems (1.13, 95% CI: 0.21, 2.06). Children from Sumgait were reported by parents to have significantly higher levels of ADHD symptomatology (3.44, 95% CI: 0.54, 6.34), SDQ total difficulties (2.46, 95% CI: 0.68, 4.25) and SDQ internalizing problems (1.72, 95% CI: 0.43, 3.01) compared to children from the capital city. Caregivers who completed full secondary education (up to grade 11) reported lower levels of total SDQ difficulties in their children than those with incomplete secondary education (-1.51, 95% CI: -2.97, -0.05). Additionally, children from larger households were reported by caregivers to have higher levels of internalizing problems (0.40, 95% CI: 0.09, 0.70).

#### Variance explained at the family level

The models indicated that around 21% of the variance in SDQ total difficulties and 18% of the variance in SDQ internalizing problems could be explained at the family level.

## Discussion

This is the first study to investigate the prevalence of mental health symptoms among children in Azerbaijan who have been reunited with their families following institutionalization and to examine their associations with various institutional and sociodemographic characteristics.

### Overall patterns of emotional wellbeing

Overall, children in this study maintained positive self-esteem and demonstrated higher prosocial skills compared to community norms on the SDQ prosocial subscale [34]. Enhanced social skills may develop through more frequent, positive, and meaningful interactions with others, and can serve as a protective factor against adversities in life. Creating educational opportunities like those offered by these residential schools for low-income families may, in turn, provide a supportive social environment for children who would otherwise have limited access to such experiences.

However, our findings also indicate a high prevalence of mental health symptoms among children who have experienced institutional placement and subsequent deinstitutionalization. Notably, while the overall prevalence rates of mental health symptoms reported by children and caregivers were comparable, the correlates of these symptoms differed across informants. Furthermore, we identified several demographic and socioeconomic factors that consistently correlate with mental health symptoms among reunified children.

Based on parent-reported SDQ total difficulties scores, 14.88% and 44.65% of children scored in ‘borderline’ and ‘abnormal’ ranges, respectively. In contrast, the community-based sample studied in Azerbaijan reported significantly lower proportions, with only 12.2% and 15.7% scoring in ‘borderline’ and ‘abnormal’ ranges, respectively, and a mean score of 10.8 (SD = 5.4) [34]. Notably, the mean scores on all four difficulties subscales of the SDQ were also higher compared to those reported in a community sample [34]. The proportion of children in our study that scored within ‘borderline’ and ‘abnormal’ ranges (per older SDQ classification) was similar to a sample of pediatric outpatients in Azerbaijan [35], and there is a higher known prevalence of mental health symptoms in pediatric primary care compared to community samples [6, 33].

Additionally, over a third of children scored above the clinical threshold for hyperactivity and inattention, suggesting a substantially higher prevalence in this population compared to community samples [9]. This finding is consistent with elevations observed on the SDQ hyperactivity subscale, highlighting attentional and behavioral regulation as key areas of vulnerability among reunified children. As these children reintegrate into regular schools, such difficulties may compromise their capacity to concentrate, follow instructions, and manage classroom demands, thereby placing them at heightened risk for academic underachievement and school disengagement.

Overall, the majority of children exhibited at least one mental health challenge above the clinical threshold, underscoring the need not only for improved screening strategies - such as using the SDQ in schools and primary care – but also effective early intervention strategies. For example, low-resource approaches, such as providing additional training for teachers and primary care providers, and incorporating similar screening tools into annual medical pediatric examinations, could considerably enhance early detection and referral for mental health disorders in this vulnerable population. Given the relatively high prevalence and cooccurrence of multiple mental health difficulties, universal preventive interventions delivered through schools and primary care may also be particularly valuable. Embedding low-cost, evidence-based prevention programs—such as classroom-wide social–emotional learning curricula, teacher training in behavior management, and parent psychoeducation—could help reduce the onset or escalation of problems and improve well-being at the population level.

### Contexts of adversity surrounding institutional care placement

Children reunifying following out-of-home placement in the U.S. are also known to have a high prevalence of internalizing and externalizing mental health symptoms [4, 23]. In the Azerbaijani context, several interrelated factors may explain the relatively high prevalence of such symptoms, including persistent family difficulties that initially led to institutionalization, household economic hardship, stress of the reunification process, and renewed exposure to unresolved family conflicts and financial challenges. A qualitative study in Azerbaijan revealed that, prior to institutional placement, children frequently endured multiple and often chronic traumatic experiences (e.g., trauma of separation, family disruptions due to domestic violence, parental divorce, parental incarceration, or parental substance abuse) [24]. These early adverse childhood experiences were further exacerbated by the institutional environment, which was often associated with feelings of abandonment, rigid routines, limited freedom and privacy, insufficient developmental stimulation, and, at times, a lack of safety. This early adversity associated with institutional care offers a potential explanation for the high prevalence of mental health symptoms found in the current study.

In addition to the institutional care experience, the post-reunification context appears to play an important role in determining mental health outcomes. Barth et al. demonstrated that reunified children with a higher likelihood of reentry tended to exhibit higher problem behavior scores as measured by the Child Behavior Checklist and they were more likely to reside in households with three or more children while living at home post-reunification [1]. The high prevalence of mental health symptoms among children in this study underscores the importance of interventions addressing mental wellbeing. Neglecting these interventions could potentially undermine the effectiveness of family reunification programs in fSU countries.

### Parent–child concurrence in reported emotional and behavioral symptoms

In this study, children’s emotional problems reported by parents align with children’s self-reported symptoms, with more than half exceeding the clinical threshold for depressive symptoms. Moreover, over a third of children reported symptoms of psychological trauma above the clinical cut-off —with avoidance symptoms surpassing intrusion symptoms—which may help explain the elevated levels of emotional difficulties, particularly those internalizing in nature (e.g., withdrawal, persistent sadness, fears, anxiety). Connell et al. demonstrated that a significant portion of children transitioning from out-of-home placement to family reunification are vulnerable to subsequent maltreatment, particularly if their entry into foster care was precipitated by maltreatment such as neglect or physical abuse [11]. Given the high prevalence of internalizing symptoms coupled with elevated PTSD symptoms, family reunification programs should prioritize screening for these symptoms and equip children and parents with the necessary skills to cope with emotional trauma and prevent them in the future.

Discrepancies between different informants’ reports of the same behavior commonly exhibit low to moderate correspondence, with stronger agreement for externalizing than for internalizing symptoms [12]. However, in this study, we did not observe the expected pattern of greater concern over externalizing symptoms or underreporting of internalizing symptoms by caregivers, possibly due to the overall severity of children’s conditions.

### Child-level developmental and institutional patterns

A child’s age emerged as an important factor for several mental health symptoms, suggesting that mental health symptomatology not only varies developmentally but may only intensify over time. Compared to elementary school age children, preadolescent-age children reported lower self-esteem and higher depression and PTSD symptoms, which positively correlated with internalizing problems based on parental reports. Gender played an important role in symptom reporting by both children and parents. Girls demonstrated a more positive self-esteem, with no significant differences in self-reported symptoms of depression or PTSD between boys and girls. However, parents reported fewer externalizing symptoms and more internalizing symptoms in girls. Previous studies have reported parent–child discrepancies in symptom reporting and their relationship to the child’s gender [21, 22]. However, further research is needed to understand how specific symptoms are perceived by parents depending on the child’s gender across different cultural and economic contexts.

Interestingly, the number of years spent in an institution did not correlate with mental health symptoms reported by either the child or the parent. Given the narrow range of institutional duration in this sample, we do not rule out the possibility that if a child were to spend significantly longer periods in institutional care than those observed here, the effects of duration might become more pronounced. Parental reports did not describe any discernible differences based on children’s birth order. Nonetheless, middle children exhibited markedly lower self-esteem in comparison to first-born children. We posit that several factors may afford greater protection to first-born children, such as a young family’s better initial economic status, reduced conflicts, and heightened attentiveness. Additionally, in Azerbaijan, first-born children traditionally receive more attention from grandparents and nurturing care from the extended family (‘*the oldest grandchild*’). However, this trend was not observed among the youngest children, who are also traditionally often afforded greater levels of protection (*a spoiled baby*’).

Consistent with previous research [38], parents of children with special needs in the current study reported increased scores for mental health symptoms on all utilized scales. However, child with physical, sensory or other special needs did not report significantly higher levels of mental health symptoms themselves.

### Caregiver characteristics

Non-biological caregivers reported fewer internalizing symptoms. Based on these findings, it can be speculated that there is a deeper mutual emotional connection between a child and their biological parents, potentially leading to a better understanding of the child’s internal experiences and a more supportive relationship. Another important consideration is the difference in how fathers and mothers perceive children’s mental well-being, with fathers reporting less symptoms. This may be a result of fathers spending fewer hours in total with their children as compared to mothers. Older caregivers reported higher levels of emotional and behavioral difficulties in children, whereas a caregiver’s age was not associated with child-reported mental health symptoms. Thus, it can be speculated that younger parents, due to less experience, may have noticed fewer symptoms or, conversely, that older parents might be less tolerant of children’s problem behaviors.

### Socioeconomic and regional disparities

Receiving public assistance was negatively associated with children’s reports of self-esteem, possibly because families receiving such assistance tend to be more socially and economically disadvantaged. Moreover, the very notion of relying on public assistance may carry social stigma, further undermining children’s sense of worth and social standing. Thus, greater care is warranted in the delivery of assistance programs to preserve children’s dignity and prevent additional marginalization. Other socioeconomic factors did not correlate with children’s reported mental health outcomes. Consistent with existing literature, poverty may compromise children’s emotional well-being; however, its effects are generally mediated through parental behaviors rather than being direct [25–27].

In contrast, socio-economic factors were significantly associated with multiple parent-reported indicators of child mental health. Caregivers’ perceived low socioeconomic status was the most robust factor positively correlated with perceived child mental health problems, particularly with externalizing problems and ADHD symptoms but not internalizing symptoms. Parental unemployment also emerged as a significant correlate of higher parent-reported hyperactivity-impulsivity symptoms in children. Consistent with the Family Stress Model, economic strain may heighten parental distress and reduce emotional tolerance, thereby affecting parents’ ability to manage children’s behavioral difficulties effectively [10, 18, 42]. Structural equation modeling in this sample supported these pathways, indicating that economic deprivation (including parental unemployment) undermines parental emotional well-being and fosters harsher parenting practices, leading to elevated emotional and behavioral problems, including children’s self-reported symptoms of depression and trauma [25].

A higher level of caregiver education was linked to fewer reported emotional and behavioral problems in children, indicating that educational attainment—a common proxy for socioeconomic status—may act as a protective factor against such difficulties.

Disparities in mental well-being among children were observed across different residence sites, with outcomes less favorable outside the capital. This pattern emerged in both child-reported (lower self-esteem and higher PTSD symptoms) and parent-reported (higher SDQ total difficulties and ADHD symptoms) outcomes, the latter having strong associations with lower socio-economic factors. It is challenging to pinpoint a single reason for this discrepancy, but it may reflect differences in city sizes, access to services and resources, economic opportunities, and social status between Baku–a large megapolis–and smaller urban centers. Another possibility is that more severe or troublesome cases are located in sites outside Baku, or that children in the capital benefit from greater availability of services and better-trained staff. These findings point to the need for more targeted attention and services for children and families in regional areas and institutions.

Larger household size was the only socioeconomic factor associated with greater parent-reported internalizing problems in this study, possibly reflecting increased economic strain and limited household resources. The relationship between household size and children’s emotional difficulties, however, is complex and varies across cultural and contextual settings [1]. On one hand, larger families may experience a dilution of parental time, attention, and emotional support, leading to reduced individualized support and greater vulnerability to internalizing symptoms. This interpretation is consistent with broader social science and economic research showing that greater family size can strain both material and emotional resources. On the other hand, larger households–with multiple caregivers or siblings–may facilitate greater observation and awareness of a child’s emotional state, increasing the likelihood that such difficulties are detected and brought to parents’ attention. Consistent with the former interpretation, Barth and colleagues [1] found that having three or more siblings increased the likelihood of reentry into institutional care, suggesting that larger family size may exacerbate stress among economically disadvantaged families already at risk of institutionalization.

## Limitations

There were several limitations in this study. First, different tools were used by children and caregivers to assess mental health symptoms. Second, the absence of clinically confirmed diagnoses limits the clinical validity of scale scores, which may indicate potential disorders but do not provide definitive diagnostic confirmation. The limited diagnostic precision of scale-derived scores may reduce the generalizability of the findings and should be considered when planning interventions. Third, there is limited information about the processes and events within institutions as well as children’s pre-institutional mental status. Therefore, it is challenging to objectively verify their prior experiences, limiting the causal interpretation of our findings. Fourth, children who underwent reunification may systematically differ from those who remained in institutional care, potentially introducing selection bias. Fifth, information regarding caregivers’ mental health status was not included in the current analysis. Sixth, the study did not include a community control group, limiting our ability to determine baseline prevalence of mental health symptoms and reducing the generalizability of our findings. Finally, due to the intricate nature of social interactions, it was not feasible to comprehensively address all socio-economic aspects, thereby rendering the variables in this study a rough approximation of the actual economic status of each individual dyad. Future research should further examine the role of risk and protective factors on individual, family, and social levels.

## Conclusions

Our findings underscore a substantial prevalence of mental health symptoms among children who have undergone family reunification following placement in institutional care. The broad spectrum of symptoms observed emphasizes the critical importance of mental health preventive interventions for this group along with early screening to ensure timely detection and intervention. We also identified notable disparities in the perception of mental health issues between children and their caregivers, highlighting the need for multi-informant approaches to maximize screening efficiency and avoid missed cases. Our investigation identified several demographic and socioeconomic factors that correlate with mental health symptoms among reunified children. These findings highlight the need for multi-level interventions that address the diverse needs and perspectives of both children and caregivers, while also incorporating developmental, gender-responsive, approaches and tackling family and structural drivers that shape mental health outcomes following deinstitutionalization and reintegration into society.

## Data Availability

The datasets analyzed in the present study are not publicly available because of the confidentiality of the participants but are available from the corresponding author upon reasonable request.

## Abbreviations

ADHD: Attention-Deficit/Hyperactivity Disorder
AZN: Azerbaijani manat
CES-D: Center for Epidemiologic Studies Depression
CI: Confidence interval
CRIES-8: Child Revised Impact of Events Scale-8
fSU: Former Soviet Union
IRB: Institutional Review Board
NICHD: Eunice Kennedy Shriver National Institute of Child Health and Human Development
PTSD: Post-traumatic stress disorder
SDQ: Strengths and Difficulties Questionnaire
SES: Socioeconomic status

## References

[1] Barth RP, Weigensberg EC, Fisher PA, Fetrow B, Green RL. Reentry of elementary aged children following reunification from foster care. Child Youth Serv Rev. 2008;30(4):353–64. 10.1016/j.childyouth.2007.10.002. 21765570 10.1016/j.childyouth.2007.10.002 PMC3134969

[2] Beard L, Ismayilova L, Claypool E, Heidorn E, Guliyeva N. From child institutions to family reunification: challenges and opportunities in Building family-centred care. Lancet Glob Health. 2022;10:S31. 10.1016/S2214-109X(22)00160-7.

[3] Baughman N, Prescott SL, Rooney R. The prevention of anxiety and depression in early childhood. Front Psychol. 2020;11:517896. 10.3389/fpsyg.2020.517896. 33101112 10.3389/fpsyg.2020.517896 PMC7554369

[4] Bellamy L. Behavioral problems following reunification of children in long-term foster care. Child Youth Serv Rev. 2008;30(2):216–28. 10.1016/j.childyouth.2007.09.008. 19190714 10.1016/j.childyouth.2007.09.008 PMC2344153

[5] Bick J, Fox N, Zeanah C, Nelson CA. Early deprivation, atypical brain development, and internalizing symptoms in late childhood. Neuroscience. 2017;342:140–53. 10.1016/j.neuroscience.2015.09.026. Epub 2015 Sep 16. PMID: 26384960; PMCID: PMC4794419. 26384960 10.1016/j.neuroscience.2015.09.026 PMC4794419

[6] Bilfield S, Wildman BG, Karazsia BT. Brief report: the relationship between chronic illness and identification and management of psychosocial problems in pediatric primary care. Pedia Psychol. 2006;31(8):813–7. 10.1093/jpepsy/jsj092. 10.1093/jpepsy/jsj092 16354938

[7] Carter R. Family matters: A study of institutional child care in central and Eastern Europe and the former Soviet union. London, UK: EveryChild; 2005.

[8] Claypool E, Ismayilova L. A gender-focused analysis of structural and social precipitators to child institutionalization in azerbaijan: A qualitative study. Soc Sci Med. 2019;232:262–9. 31108331 10.1016/j.socscimed.2019.04.039

[9] Coghill D, Seth S. Effective management of attention-deficit/hyperactivity disorder (ADHD) through structured re-assessment: the Dundee ADHD clinical care pathway. Child Adoles Psychia Ment Heal. 2015;9(1):1–14. 10.1186/s13034-015-0083-2. 10.1186/s13034-015-0083-2 PMC4652349 26587055

[10] Conger RD, Conger KJ, Martin MJ. Socioeconomic status, family processes, and individual development. J Marriage Fam. 2010;72(3):685–704. 10.1111/j.1741-3737.2010.00725.x. 10.1111/j.1741-3737.2010.00725.x PMC2910915 20676350

[11] Connell CM, Vanderploeg JJ, Katz KH, Caron C, Saunders L, Tebes JK. Maltreatment following reunification: predictors of subsequent child protective services contact after children return home. Child Abuse Negl. 2009;33(4):218–28. 10.1016/j.chiabu.2008.07.005. 19327834 10.1016/j.chiabu.2008.07.005 PMC3867131

[12] De Los Reyes A. Strategic objectives for improving Understanding of informant discrepancies in developmental psychopathology research. Dev Psychopathol. 2013;25(03):669–82. 10.1017/s0954579413000096. 23880384 10.1017/S0954579413000096

[13] Desmond CK, Watt K, Saha A, Huang J, Lu C. Prevalence and number of children living in institutional care: global, regional, and country estimates. Lancet Child Adolesc Health. 2020;4(5):370–7. 10.1016/S2352-4642(20)30022-5. 32151317 10.1016/S2352-4642(20)30022-5

[14] DuPaul GJ, Power TJ, Anastopoulos AD, Reid R. ADHD rating Scale—IV: Checklists, norms, and clinical interpretation. Guilford Press; 1998.

[15] Erol N, Simsek Z, Münir K. Mental health of adolescents reared in institutional care in turkey: challenges and hope in the twenty-first century. Eur Child Adolesc Psychiatry. 2010;19(2):113–24. 10.1007/s00787-009-0047-2. Epub 2009 Jul 31. PMID: 19644732; PMCID: PMC3124379. 19644732 10.1007/s00787-009-0047-2 PMC3124379

[16] Faulstich ME, Carey MP, Ruggiero L, et al. Assessment of depression in childhood and adolescence: an evaluation of the center for epidemiological studies depression scale for children (CES-DC). Am J Psychiatry. 1986;143(8):1024–7. 10.1176/ajp.143.8.1024. 3728717 10.1176/ajp.143.8.1024

[17] Fitts WH, Warren WL. Tennessee Self-Concept scale: TSCS-2. Los Angeles: Western Psychological Services; 1996. p. 118.

[18] Gautam N, Rahman MM, Hashmi R, Lim A, Khanam R. Socioeconomic inequalities in child and adolescent mental health in australia: the role of parenting style and parents’ relationships. Child Adolesc Psychiatry Ment Health. 2024;18(1):28. 10.1186/s13034-024-00719-x. 10.1186/s13034-024-00719-x PMC10882797 38383394

[19] Goodman R Psychometric properties of the Strengths and Difficulties Questionnaire. J Am Acad Child Adolesc Psychiatry. 2001;40(11):1337–45. . 10.1097/00004583-200111000-00015. 11699809 10.1097/00004583-200111000-00015

[20] Gray CL, Pence BW, Ostermann J, Whetten RA, O’Donnell K, Thielman NM, Whetten K. Prevalence and incidence of traumatic experiences among orphans in institutional and Family-Based settings in 5 Low- and Middle-Income countries: A longitudinal study. Glob Health Sci Pract. 2015;3(3):395–404. 10.9745/GHSP-D-15-00093. PMID: 26374801; PMCID: PMC4570014. 26374801 10.9745/GHSP-D-15-00093 PMC4570014

[21] Gray EJ, Scott JG, Lawrence DM, Thomas HJ. Concordance between adolescents and parents on the strengths and difficulties questionnaire: analysis of an Australian nationally representative sample. Aust N Z J Psychiatry. 2021;55(11):1058–70. Epub 2021 Apr 30. PMID: 33926246.10.1177/00048674211009610. 10.1177/00048674211009610 33926246

[22] Hen M, Shenaar-Golan V, Atia S, Yatzkar U. Child-parent agreement on the SDQ: the role of child-parent attachment and parental feelings. J Clin Psychol. 2024;80(9):2045–62. 10.1002/jclp.23707. Epub 2024 May 29. PMID: 38809521. 38809521 10.1002/jclp.23707

[23] Ismayilova FS, Aytakin H. Reforming child institutional care in the post-Soviet bloc: the potential role of family-based empowerment strategies. Child Youth Serv Rev. 2014;47(2):136–48. 10.1016/j.childyouth.2014.09.007.

[24] Ismayilova L, Claypool E, Heidorn E. Trauma of separation: the social and emotional impact of institutionalization on children in a post-soviet country. BMC Public Health. 2023;23:366. 10.1186/s12889-023-15275-w. 36803447 10.1186/s12889-023-15275-w PMC9942302

[25] Ismayilova L, Fu L, Salayev K, Wang SH, Heidorn E. Pathways to child mental health: A structural equation model of economic Deprivation, Parenting, and internalizing problems. SSM-Mental Health. 2025;9:100528. 10.1016/j.ssmmh.2025.100528. 10.1016/j.ssmmh.2025.100528 PMC12652412 41311713

[26] Kaiser T, Li J, Pollmann-Schult M, Song AY. Poverty and child behavioral problems: the mediating role of parenting and parental well-being. Int J Environ Res Public Health. 2017;14(9):981. 10.3390/ijerph14090981. 10.3390/ijerph14090981 PMC5615518 28867777

[27] Karimli L, Ismayilova L, Wells CR. Pathways from poverty to child mental health in Burkina faso: longitudinal mediation analyses in a Cluster-Randomized clinical trial. J Adolesc Health. 2025;76(3):415–28.10.1016/j.jadohealth.2024.10.030. 10.1016/j.jadohealth.2024.10.030 39692681

[28] Lau AS. Going home: the complex effects of reunification on internalizing problems among children in foster care. J Abnorm Child Psychol. 2003;31(4):345–58. 10.1023/a:1023816000232. 12831225 10.1023/a:1023816000232

[29] Nsabimana E, Rutembesa E, Wilhelm P, Martin-Soelch C. Effects of institutionalization and parental living status on children’s Self-Esteem, and externalizing and internalizing problems in Rwanda. Front Psychiatry. 2019;10:442. 10.3389/fpsyt.2019.00442. PMID: 31275183; PMCID: PMC6593105. 31275183 10.3389/fpsyt.2019.00442 PMC6593105

[30] Pawliczuk W, Kaźmierczak-Mytkowska A, Srebnicki T, Wolańczyk T. The prevalence of mental disorders among children and youth staying in residential institutions, children’s homes - a review of epidemiological studies. Psychiatr Pol. 2018;52(2):345–53. 10.12740/PP/75738. English, Polish. 29975371 10.12740/PP/75738

[31] Perrin S, Meiser-Stedman R, Smith P. The children’s revised impact of event scale (CRIES): validity as a screening instrument for PTSD. Behav Cogn Psychothe. 2005;33(4):487–98. 10.1017/S1352465805002419.

[32] Petrowski N, Cappa C, Gross P. Estimating the number of children in formal alternative care: challenges and results. Child Abuse Negl. 2017;70:388–98. 10.1016/j.chiabu.2016.11.026. 28578826 10.1016/j.chiabu.2016.11.026

[33] Polaha J, Dalton WT, Allen S. The prevalence of emotional and behavior problems in pediatric primary care serving rural children. J Pediatr Psychol. 2011;36(6):652–60. 10.1093/jpepsy/jsq116. 21227909 10.1093/jpepsy/jsq116

[34] Salayev KA, Sanne B, Salayev R. Psychiatric and behavioural problems in children and adolescents with epilepsy. East Asian Arch Psychiatry. 2017;27(3):106–14. 28993543

[35] Salayev K, Rustamov I, Gadjiyeva N, Salayev R, Sanne B. The discriminative ability of the Azeri version of the strengths and difficulties questionnaire in outpatient practice. Community Ment Health J. 2016;52(8):1106–12. 10.1007/s10597-014-9791-y. 25535043 10.1007/s10597-014-9791-y PMC4478281

[36] Skinner A, Occhipinti JA, Song YJC, Hickie IB. Population-level effectiveness of alternative approaches to preventing mental disorders in adolescents and young adults. Sci Rep. 2023;13(1):19982. 10.1038/s41598-023-47322-2. PMID: 37968445; PMCID: PMC10652005. 37968445 10.1038/s41598-023-47322-2 PMC10652005

[37] Snijders TA, Bosker R. Multilevel analysis: an introduction to basic and advanced multilevel modeling. London etc.: Sage; 2012. p. 368.

[38] Täljedal T, Granlund M, Almqvist L, Osman F, Norén Selinus E, Fängström K. Patterns of mental health problems and well-being in children with disabilities in sweden: A cross-sectional survey and cluster analysis. PLoS ONE. 2023;18(7):e0288815. 10.1371/journal.pone.0288815. 37463139 10.1371/journal.pone.0288815 PMC10353824

[39] U.S. National Institute of Mental Health. Grand Challenges in Global Mental Health. Accessed 18 Oct 2025. https://www.nimh.nih.gov/about/organization/cgmhr/grandchallenges.

[40] Ulybina O Explaining the cross-national pattern of policy shift toward childcare deinstitutionalization. Int J Sociol. 202;52(2):128–55. 10.1080/00207659.2022.2031488.

[41] Van IJzendoorn MH, Bakermans-Kranenburg MJ, Coughlan B, Reijman S. Annual research review: umbrella synthesis of meta-analyses on child maltreatment antecedents and interventions: differential susceptibility perspective on risk and resilience. J Child Psychol Psychia. 2019;61(3):272–90. 10.1111/jcpp.13147. 10.1111/jcpp.13147 PMC7065145 31667862

[42] Wang Y, McLeroy AM. Poverty, parenting stress, and adolescent mental health: the protective role of school connectedness reexamined. Child Youth Serv Rev. 2023;153:107127. 10.1016/j.childyouth.2023.107127.

[43] Wiik KL, Loman MM, Van Ryzin MJ, Armstrong JM, Essex MJ, Pollak SD, Gunnar MR. Behavioral and emotional symptoms of post-institutionalized children in middle childhood. J Child Psychol Psychiatry. 2011;52(1):56–63. PMID: 20649913; PMCID: PMC2978793. 20649913 10.1111/j.1469-7610.2010.02294.x PMC2978793

[44] Wulczyn F. Family reunification. Future Child. 2004;14(1):95–113. 10.2307/1602756. 15072020

[45] Zewude B, Tadele G, Engdawork K, Assefa S. A social-ecological view of the factors affecting the effectiveness of reintegration interventions targeting children out of family-based care situations: A scoping review. Cogent Soc Sci. 2023;9(2). 10.1080/23311886.2023.2277343.

